# Magnitude processing of symbolic and non-symbolic proportions: an fMRI study

**DOI:** 10.1186/s12993-018-0141-z

**Published:** 2018-05-10

**Authors:** Julia Mock, Stefan Huber, Johannes Bloechle, Julia F. Dietrich, Julia Bahnmueller, Johannes Rennig, Elise Klein, Korbinian Moeller

**Affiliations:** 1 Leibniz-Institut für Wissensmedien, Schleichstraße 6, 72076 Tuebingen, Germany; 2 Division of Neuropsychology, Hertie-Institute for Clinical Brain Research, Otfried-Müller-Straße 27, 72076 Tuebingen, Germany; 3 Eberhardt-Karls University Tuebingen, 72074 Tuebingen, Germany

**Keywords:** Proportions, Fractions, Decimals, Magnitude processing, fMRI

## Abstract

**Background:**

Recent research indicates that processing proportion magnitude is associated with activation in the intraparietal sulcus. Thus, brain areas associated with the processing of numbers (i.e., absolute magnitude) were activated during processing symbolic fractions as well as non-symbolic proportions. Here, we investigated systematically the cognitive processing of symbolic (e.g., fractions and decimals) and non-symbolic proportions (e.g., dot patterns and pie charts) in a two-stage procedure. First, we investigated relative magnitude-related activations of proportion processing. Second, we evaluated whether symbolic and non-symbolic proportions share common neural substrates.

**Methods:**

We conducted an fMRI study using magnitude comparison tasks with symbolic and non-symbolic proportions, respectively. As an indicator for magnitude-related processing of proportions, the distance effect was evaluated.

**Results:**

A conjunction analysis indicated joint activation of specific occipito-parietal areas including right intraparietal sulcus (IPS) during proportion magnitude processing. More specifically, results indicate that the IPS, which is commonly associated with absolute magnitude processing, is involved in processing relative magnitude information as well, irrespective of symbolic or non-symbolic presentation format. However, we also found distinct activation patterns for the magnitude processing of the different presentation formats.

**Conclusion:**

Our findings suggest that processing for the separate presentation formats is not only associated with magnitude manipulations in the IPS, but also increasing demands on executive functions and strategy use associated with frontal brain regions as well as visual attention and encoding in occipital regions. Thus, the magnitude processing of proportions may not exclusively reflect processing of number magnitude information but also rather domain-general processes.

## Background

Fractions, ratios, and proportions are among the most ubiquitous forms of numerical information encountered in everyday life. Yet, they are also one of the most difficult concepts to learn and even adults frequently fail to process them correctly [1, 2]. Therefore, understanding the processing and acquisition of fractions and proportions poses one of the most challenging problems in numerical cognition research as well as mathematics education [3].

In teaching and learning fractions, symbolic and non-symbolic presentation formats are often presented side by side to successfully foster conceptual understanding of proportional relations [4–6]. The present study aims at exploring why these pedagogic approaches might be successful from a neurocognitive perspective. To this end, we aimed at broadening the understanding of mechanisms underlying proportion processing by investigating the neural correlates of processing symbolic fractions and non-symbolic proportions in the human brain. In particular, a shared neural correlate for the magnitude processing of fractions and proportions, independent of their presentation format, might explain the efficacy of these pedagogic approaches.

Before the details of the current study will be outlined, we will give a brief summary of recent advances in numerical cognition research by describing (i) neural networks involved in number processing in general, (ii) processes of symbolic and non-symbolic quantities and their underlying neural correlates in particular, and (iii) argue how our investigation of a common neural substrate for both symbolic and non-symbolic proportion processing can be informative for a better understanding of relative magnitude processing.

### Neural networks involved in number processing

Previous studies on number processing showed that the intraparietal sulcus (IPS) is crucially involved in the processing of absolute quantity and number magnitude [7–10]. To evaluate the processing of magnitude information conveyed by natural numbers and fractions, the numerical distance effect in magnitude comparison tasks has been employed repeatedly. The numerical distance effect reflects the finding of shorter and more accurate responses with larger numerical distance between two to-be-compared numbers (e.g., 1_9 vs. 4_5; [11]). Importantly, the presence of the numerical distance effect is considered to indicate number magnitude processing in the task at hand [11, 12].

Behavioral results on the distance effect were substantiated by findings showing that activation within the IPS was negatively correlated with numerical distance in number magnitude comparison tasks for natural numbers (e.g., [13], but see [14]). This indicates that the IPS seems to play a crucial role in the representation and processing of number magnitude information [13–17].

However, although neuroimaging research on number processing primarily focused on parietal cortex and especially on the IPS, a rather complex system of functional brain networks was observed to contribute to numerical cognition in general [18, 19]. Besides the IPS, numerical distance was also shown to negatively correlate with activation in bilateral prefrontal and precentral cortex, indicating fronto-parietal networks of number magnitude processing [9, 20]. However, recent research suggests an even broader network to be involved in numerical cognition.

For instance, there is evidence that early perceptual numerical features are decoded in the ventral visual stream, including V1 and the inferior temporal cortex (ITC), before visual-spatial features of numerical quantity are processed in the IPS and the superior parietal lobule (SPL; [18, 21]). Moreover, it was suggested that a widespread fronto-parietal network, comprising IPS, supramarginal gyrus, supplementary motor areas, and dorsolateral prefrontal cortex (DLPFC), is involved in planning, executing, and monitoring arithmetic procedures as well as maintaining intermediate results [18, 22–24]. Additionally, DLPFC as well as anterior cingulate cortex (ACC) were also associated with processes of cognitive control to optimize performance by monitoring and adapting task execution as well as inhibiting undesired responses [18, 25–27]. Furthermore, the angular gyrus (AG) was also argued to be involved in verbal retrieval of math facts ([10, 28, 29], but see [15, 30]). Finally, the anterior insula and ventrolateral prefrontal cortex were suggested to be involved in processes of guiding and maintaining goal-directed attention [18, 19].

Thus, although parietal regions, and in particular the IPS, play a central role in numerical cognition, there is growing evidence that cognitive processes such as working memory, cognitive control, and executive functions associated with frontal, temporal, and insular cortex are also vital to access numerical information, employ representations of numerical knowledge, and manipulate quantities during calculations.

### Neural processing of symbolic numbers and non-symbolic quantities

While the IPS is thought to comprise a notation-independent representation of the magnitude information conveyed by numerals [20, 31], words [9, 32], or non-symbolic arrays as quantities [33, 34], Sokolowski and colleagues [35] observed several additional areas jointly activated in processing symbolic as well as non-symbolic quantities. As a result of a meta-analysis, the authors reported joint activation of bilateral inferior parietal lobule (IPL) and precuneus as well as left superior parietal lobule (SPL) and right superior frontal gyrus (SFG) during the processing of both symbolic and non-symbolic numbers. Furthermore, Holloway and colleagues [36] reported a right-sided dominance of joint processing of symbolic and non-symbolic magnitude in right IPL and SPL. Several other studies also indicated that this region is involved in processing symbolic [9, 20, 31, 37] and non-symbolic numerical magnitude [8, 33, 38]. Furthermore, Holloway and colleagues [36] found joint activations for symbolic and non-symbolic magnitude in the inferior frontal gyrus (IFG) extending to middle frontal gyrus, right anterior insula, ACC, and SFG. Thereby, these findings imply that these brain regions comprise format-independent processing of symbolic and non-symbolic magnitudes.

However, recent research also indicated that symbolic and non-symbolic magnitudes are processed by both overlapping but also distinct neural systems [8, 35, 36]. The processing of non-symbolic magnitude was observed to involve visual cortex areas due to greater visual demands such as the individuation and summation of non-symbolic items [36]. The meta-analysis of Sokolowski and colleagues [35] revealed a right-lateralized fronto-parietal network including right SPL, IPL, precuneus, SFG, and insula as well as middle occipital gyrus involved in non-symbolic number processing compared to symbolic numbers.

In contrast, stronger activation for processing symbolic compared to non-symbolic numbers was found in right supramarginal gyrus, IPL, and left AG. Holloway and colleagues [36] also reported involvement of left AG as well as superior temporal gyrus during symbolic compared to non-symbolic number processing. These regions have repeatedly been reported to be important during exact calculation [28, 34] and arithmetic fact retrieval [29, 39].

Thus, previous research suggests that the human brain seems to represent numerical magnitude both format-dependent as well as format-independent, and thus, abstract [35].

### Neural correlates of processing symbolic fractions and non-symbolic proportions

Recent studies indicated that the same brain regions associated with processing absolute magnitude are also involved in processing fractions and proportions, and thus, relative magnitude in general [40–43]. Importantly, the magnitude of a fraction (e.g., ¼) might be represented by the numerical magnitude of the fraction as a whole (e.g., .25) or involve separate representations of the magnitudes of numerator and denominator. Ischebeck and colleagues [41] found that activation within the right IPS, right medial frontal gyrus, and middle occipital gyrus was only modulated by the overall numerical distance between fractions and was not influenced by numerator or denominator distances. Therefore, these authors concluded that fraction magnitude is represented holistically at the neural level.

Moreover, Jacob and Nieder [42] provided evidence that the processing of fraction magnitude within the IPS seems to be independent of presentation format. Using a functional MRI adaptation (fMRA) paradigm, participants were habituated to a given fraction number (e.g., $${\raise0.7ex\hbox{$1$} \!\mathord{\left/ {\vphantom {1 6}}\right.\kern-0pt} \!\lower0.7ex\hbox{$6$}}$$) and were then presented with either a deviant fraction number (e.g., ½) or fraction word (e.g., ‘one-half’). During adaptation, the blood oxygen level-dependent (BOLD) signal decreased. When presented with deviants, signal in bilateral IPS, bilateral prefrontal cortex, and a small cluster in the right cingulate cortex recovered as a function of numerical distance between deviant and adapted fraction magnitude. This effect was independent of presentation format. This suggests that the same populations of neurons seem to code the same fraction magnitude, irrespective of presentation format.

Jacob and Nieder [43] also observed that the BOLD signal in bilateral IPS and lateral prefrontal cortex decreased during the adaptation phase in an fMRA experiment using non-symbolic proportions (e.g., proportions of line lengths or numerosities). Again, BOLD signal recovered when presented with a deviant stimulus as a function of the distance between the deviant and the adapted proportion with strongest effects in bilateral anterior IPS. Further clusters of activations were found in bilateral prefrontal and precentral regions with seemingly right-lateralized dominance.

Taken together, previous work indicates that a network comprising bilateral IPS, prefrontal cortex, middle occipital gyrus, and cingulate cortex, which was reported to be activated for processing absolute numerical magnitude, is also activated when relative magnitude needs to be processed, irrespective of presentation format (for a brief overview see [13]).

### The present study

So far, a common neural substrate for processing proportion magnitude was observed only for (i) symbolic fractions and fraction words [42], (ii) proportional line lengths and non-symbolic numerosities [43], and (iii) different pairs of symbolic fractions ([41], e.g., same denominator: 2/7 vs. 5/7; same nominator: 3/5 vs. 3/8; mixed pairs: 2/3 vs. 1/5). Thus, it has not yet been investigated systematically whether both symbolic and non-symbolic proportions have a common neural substrate for relative magnitude processing reflected by shared activation for processing relative magnitude independent of presentation format. However, this is an important question: in teaching and learning settings, symbolic and non-symbolic presentation formats of fractions and proportions are often used side by side to introduce and foster the understanding of proportional relations. To allow for a better and easier-to-grasp conceptual understanding of proportionality aspects, symbolic fractions in particular are often presented and illustrated using non-symbolic pie charts and proportional dot patterns [4, 5, 44–47]. Additionally, understanding of fraction magnitude is usually supported by references to its respective equivalent in decimal notations [48]. Furthermore, non-symbolic proportions can be displayed either discretely involving countable units such as patterns of, for instance, blue and yellow dots or continuously without segmentation as in pie charts to support the conceptual understanding of fractions. Therefore, the current study aimed at investigating whether magnitude processing of symbolic and non-symbolic proportions has a common neural substrate. We conducted an fMRI study using magnitude comparison tasks with symbolic (e.g., fractions and decimals) and non-symbolic proportions (e.g., dot patterns and pie charts), respectively.

As an indicator of magnitude-related processing, we specifically considered the numerical distance effect in our analyses. In a two-stage procedure, we first evaluated distance-related activations in proportion processing in different formats before addressing the issue of a common neural substrate underlying both symbolic and non-symbolic proportion processing.

Because of the similarity of decimals to integers, we expected activation in areas typically associated with the processing of symbolic numbers for the processing of decimals. These areas involve bilateral IPS, left AG, and supramarginal gyrus [21, 35, 36]. Additional to activations in bilateral IPS, we expected stronger frontal activations in SMA, DLPFC, and ACC for the processing of fraction magnitude due to higher cognitive and working memory demands reflecting additional computations necessary for accessing fraction magnitude [18, 25, 26, 41]. For proportions reflected by dot patterns, comparable cognitive and working memory demands were expected, and thus, activations in frontal areas such as DLPFC and ACC in addition to IPS [43]. Furthermore, we hypothesized that dot patterns should elicit stronger activations in visual-occipital areas because of higher visual demands as well as right IPS due to their non-symbolic nature [8, 33, 36, 38]. For pie charts, we expected activations in a fronto-parietal network including SMA, DLPFC and IPS as well as in occipital brain regions due to necessary visual processing and evaluations of part-whole relations as well as the resulting working memory demands.

As all previous studies on processing fractions or non-symbolic proportions showed an involvement of bilateral intraparietal cortex with a right-lateralized preference as well as activations in PFC, we also expected to find joint magnitude-related fronto-parietal activation in bilateral IPS and PFC for all four presentation formats.

## Methods

### Participants

Twenty-four right-handed volunteers (13 female, mean age = 23.2 years; *SD *= 2.99 years) participated in the study. All participants were university students. After being informed about the experimental procedure, they gave their written consent in accordance with the protocol of the local Ethics Committee of the Medical Faculty of the University of Tuebingen. All participants reported normal or corrected to normal vision and no previous history of neurological or psychiatric disorders. They received monetary compensation for their participation.

### Design and procedure

**Fig. 1:**
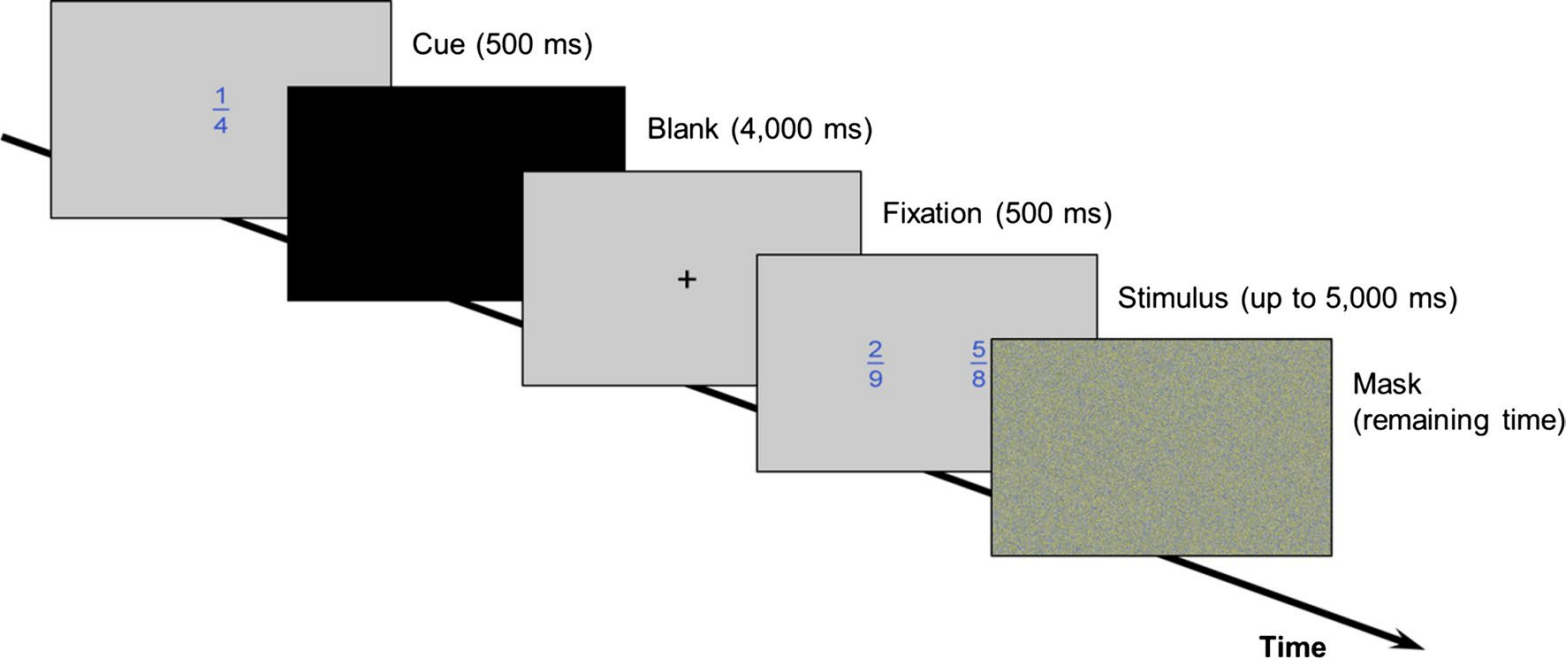
Illustration of the experimental procedure at the beginning of each block (i.e., one out of five trials)

We employed a block design with alternating comparison task blocks in four conditions (i.e., fraction, decimal, pie chart, dot pattern comparison tasks). Blocks were presented in pseudo-random order. In total, we ran 24 blocks (six blocks per condition) consisting of one practice trial and four critical trials each. Thus, the experiment consisted of six practice and 24 experimental trials per condition (24 practice and 96 experimental trials in total). Each task block was built as follows: at the beginning of each block, a cue indicating the upcoming proportion type for the next five trials was presented for 500 ms. Subsequently, a black screen was presented for 4000 ms. The cue was the fraction 1/4 shown in the different presentation formats in the center of the screen against grey background. Afterwards, critical trials were presented starting with a black fixation cross against grey background for 500 ms, followed by the presentation of a proportion stimulus for up to 5000 ms. Participants had to respond within this time limit by pressing one of two MRI compatible response buttons with either their left (indicating left proportion larger) or right thumb (indicating right proportion larger). When participants responded faster than the given 5000 ms, a mask was presented in the remaining time (visual noise consisting of blue, yellow, and grey pixels). Then the next trial was presented. The procedure of the beginning of a block is shown in Fig. 1. There was no jitter between successive stimuli. At the end of each block, a black screen was shown for 6000 ms.

### Stimuli

**Fig. 2:**
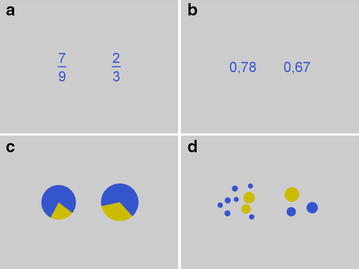
Example stimuli (7/9 vs. 2/3) for the four different presentation formats. **a** Fractions, **b** decimals, **c** pie charts and **d** dot patterns

We applied four different presentation formats of proportions: fractions, decimals, pie charts, and dot patterns (see Fig. 2). For each of these four presentation formats, we constructed 30 items. Proportions were presented in pairs with the magnitude of the first proportion ranging from .13 to .86 and of the second proportion ranging from .22 to .89. Absolute distances between proportions ranged from .02 to .69.

We first generated the symbolic fraction items and converted them into the other presentation formats. Numerators of the fractions ranged from 1 to 8 and denominators from 2 to 9. Fractions were constructed such that in half of the items the comparison of numerators and denominators was either congruent or incongruent with the comparison of overall fraction magnitude. In this context, congruency means that separate comparisons of numerator and denominator magnitudes yielded the same answer as the comparison of the overall magnitudes of fraction pairs (e.g., 1/5 < 2/9 with 1 < 2 and 5 < 9). In incongruent pairs, separate comparisons of numerator and denominator magnitudes yielded opposing answers as compared to the overall magnitude of the fractions (e.g., 5/9 < 2/3, but 5 > 2 and 9 > 3). Hence, participants could not solve the task correctly, when relying on the magnitude of numerators or denominators only. In the next step, we constructed decimals by dividing numerators by denominators and rounding up the result to two digits after the decimal mark. Fractions as well as decimals were presented in blue (RGB-values: 53, 85, 204; font type: Arial; font size: 80) on a grey background (RGB-values: 204, 204, 204). One proportion was located on the left half (x/y-coordinates: 356/384 px), whereas the other one was located on the right half (x/y-coordinates: 668/384 px) of the screen (screen resolution: 1024 ×  px).

Pie charts were drawn by dividing circles into two pie segments according to the magnitude of the respective fraction items. For instance, 5/9 was drawn by coloring 5/9 of the pie in blue (same blue as for fractions) and 4/9 in yellow (RGB-values: 203, 187, 0). The same grey as for fractions and decimals was used as a background color. Moreover, the location of the yellow part varied pseudo-randomly. We varied the size of the circles such that in half of the items the larger proportion was also larger according to the visual area of the blue pie segment, whereas in the other half of the items it was smaller. Thereby, we ensured that participants could not select the larger proportion by relying only on the visual area of pie segments. The diameter of pies ranged from 95 to 289 px.

Dot patterns were drawn on an invisible rectangular area of size 491 × 363 px in the center of the left and the right side of the screen. Location of dots was varied randomly in these invisible rectangular areas. Diameter of dots varied randomly from 21 to 98 px. Dot patterns were colored according to the fractions they denoted using the same colors as for pie charts. For instance, the dot pattern of 5/9 was drawn by coloring five dots in blue and four dots in yellow (and thus, 5 out of 9 dots were colored in blue). Moreover, we equated the sum of the yellow and blue areas of the dots across the two dot patterns which had to be compared to ensure that participants could not rely on visual area when comparing the dot patterns.

### fMRI data acquisition

MRI data were acquired using a 3T Siemens Magnetom TrioTim MRI system (Siemens AG, Erlangen, Germany). A high resolution T1-weighted anatomical scan (TR = 2300 s, matrix = 256 × 256, 176 slices, voxel size = 1.0 × 1.0 × 1.0 mm^3^; FOV = 256 mm^2^, TE = 2.92 ms; flip angle = 8°) was collected at the end of the experimental session. All functional measurements covered the whole brain using standard echo-planar-imaging (EPI) sequences (TR = 2400 ms; TE = 30 ms; flip angle = 80°; FOV = 220 mm^2^, 88 × 88 matrix; 42 slices, voxel size = 2.5 × 2.5 × 3.0 mm^3^, gap = 10%).

FMRI data was acquired in a single run. Total scanning time was approximately 20 min. We included pauses between blocks in which a black screen was presented for 6000 ms.

### Behavioral data analysis

We analyzed both reaction times and accuracy. A first inspection of the distribution of reaction times showed that they were strongly skewed to the right. To approach normal distribution while conserving statistical power, we used the inverse transformation and transformed reaction times into speed with measurement unit 1/sec [49].

We analyzed speed by running a linear mixed effects model (LME) and accuracy by running a generalized linear mixed model (GLME) with logit as link function and assuming a binomial error distribution. We ran (G)LME instead of analysis of variances (ANOVA) to be able to include random effects for both, participants and items to take into account that besides drawing only a sample of participants, we also included only a sample of all possible items [50]. Moreover, running ANOVA on accuracy (or error data) can result in spurious effects [51]. In the LME, we included fixed effects of condition (fractions, decimals, pie charts, and dot patterns) and distance between proportions as well as their interaction, random intercepts for participants as well as items (crossed random effects), and a random slope for condition (i.e., a maximal model; [52]). In the GLME, we included the same fixed effects and random intercepts for participants and items. Moreover, we effect-coded the predictor condition and centered the continuous predictor distance.

We considered only correctly solved trials in the analysis of speed. Additionally, we removed trials with absolute z-scaled residuals of the full model larger than ± 3. In total, we considered 82.6% of all trials for the analysis of speed.

Statistical analyses were run using R [53] and the R package lme4 for running (G)LME [54]. *p* values for fixed effects of LME were calculated running *F* tests using the Kenward–Roger approximation for degrees of freedom [55]. For GLME, we ran likelihood ratio tests (LRT). These methods are available via the R package afex [56]. Post-hoc tests were run using the R package multcomp [57] and corrected for multiple testing using the false discovery rate procedure by Benjamini and Hochberg [58].

### fMRI data analysis

fMRI data analysis was performed using SPM12 (http://www.fil.ion.ucl.ac.uk/spm). Images were slice-time corrected, motion corrected, and realigned to each participant’s mean image. Motion parameters did not exceed 2.5 mm translation in total (i.e., they did not exceed voxel size) and a head rotation of 1.5 degree in pitch, roll, and yaw in total. Therefore, none of the participants had to be excluded from the analyses because of head movements. The mean image was co-registered with the whole-brain volume. Imaging data was then normalized into standard stereotaxic MNI space (Montreal Neurological Institute, McGill University, Montreal, Canada). Images were resampled every 2.5 mm using 4th degree spline interpolation to obtain isovoxel and then smoothed with a 8 mm full-width half-maximum (FWHM) Gaussian kernel to accommodate inter-subject variation in brain anatomy and to increase signal-to-noise ratio in the images. The data were high-pass filtered (128 s) to remove low-frequency noise components and corrected for autocorrelation assuming an AR(1) process.

The onsets of the four presentation formats (i.e., fractions, decimals, pie charts, dot patterns) were entered as separate conditions in the GLM. As regressors of interest, logarithmic overall distance as first and reaction times as second parametric modulation of the conditions were added on the single-participant level. We decided to use overall distance (instead of reaction times) as the first parametric modulator due to its specific numerical features. Parametric modulators are serially orthogonalised in SPM. Therefore, only variance not explained by the first modulator can be explained by the second modulator. Consequently, logarithmic distance entered the model first, because its inherent numerical quality was of particular interest. Generally, no supra-threshold activation was found for the parametric modulation of RT unless stated otherwise. Movement parameters estimated at the realignment stage of preprocessing were included as covariates of no interest. Brain activation was convolved over all experimental trials with the canonical haemodynamic response function (HRF) as implemented in SPM12 and its time and dispersion derivatives.

We performed a three-stage analysis. First, we evaluated activation associated with the distance effect in all four presentation formats, respectively, to examine specific magnitude-related brain activation in proportion processing. Second, in an exploratory analysis, we examined format-specific activations of both symbolic and non-symbolic relative magnitudes. Third, analogous to previous studies on proportion processing [42, 43], a conjunction analysis was calculated as implemented in SPM12 (conjunction null, see [59]) to identify brain activation common in all four presentation formats during magnitude processing.

The SPM Anatomy Toolbox [60], available for all published cytoarchitectonic maps (http://www.fz-juelich.de/ime/spm_anatomy_toolbox), was used for anatomical localization of effects where applicable. In areas not yet implemented, the anatomical automatic labelling tool (AAL) in SPM12 (http://www.cyceron.fr/web/aalanatomical_automatic_labeling.html) was used.

If not stated otherwise, thresholds for statistical inference were set at FWE-corrected *p* < .05 at the voxel level, corrected for multiple comparisons at the cluster level to FWE-corrected *p* < .05 with a cluster size of *k *= 10 voxels.

An uncorrected statistical threshold of *p *< .001 was chosen for the conjunction analysis because four conditions of interest needed to significantly modulate the fMRI signal in a given region in the conjunction analysis. The effective *p* value for a conjunction analysis is the square of the *p* values for each component. Therefore, a more liberal threshold for such a conservative statistical procedure is justified [36].

## Results

### Behavioral results

Mean speed of participants in the four conditions for fractions, decimals, pie charts, and dot patterns, respectively, was: *M*_*fractions*_ = .57 (*SD *= .15) items/sec, *M*_*decimals*_ = 1.14 (*SD *= .17) items/sec, *M*_*pies*_ = .91 (*SD *= .17) items/sec, and *M*_*dots*_ = .60 (*SD *= .20) items/sec. Moreover, mean accuracy in the four conditions for fractions, decimals, pie charts, and dot patterns, respectively, were: *M*_*fractions*_= 81.1% (*SD *= 10.9%), *M*_*decimals*_ = 99.2% (*SD *= 1.5%), *M*_*pies*_ = 91.5% (*SD *= 4.8%), and *M*_*dots*_ = 72.5% (*SD *= 12.6%).

**Fig. 3:**
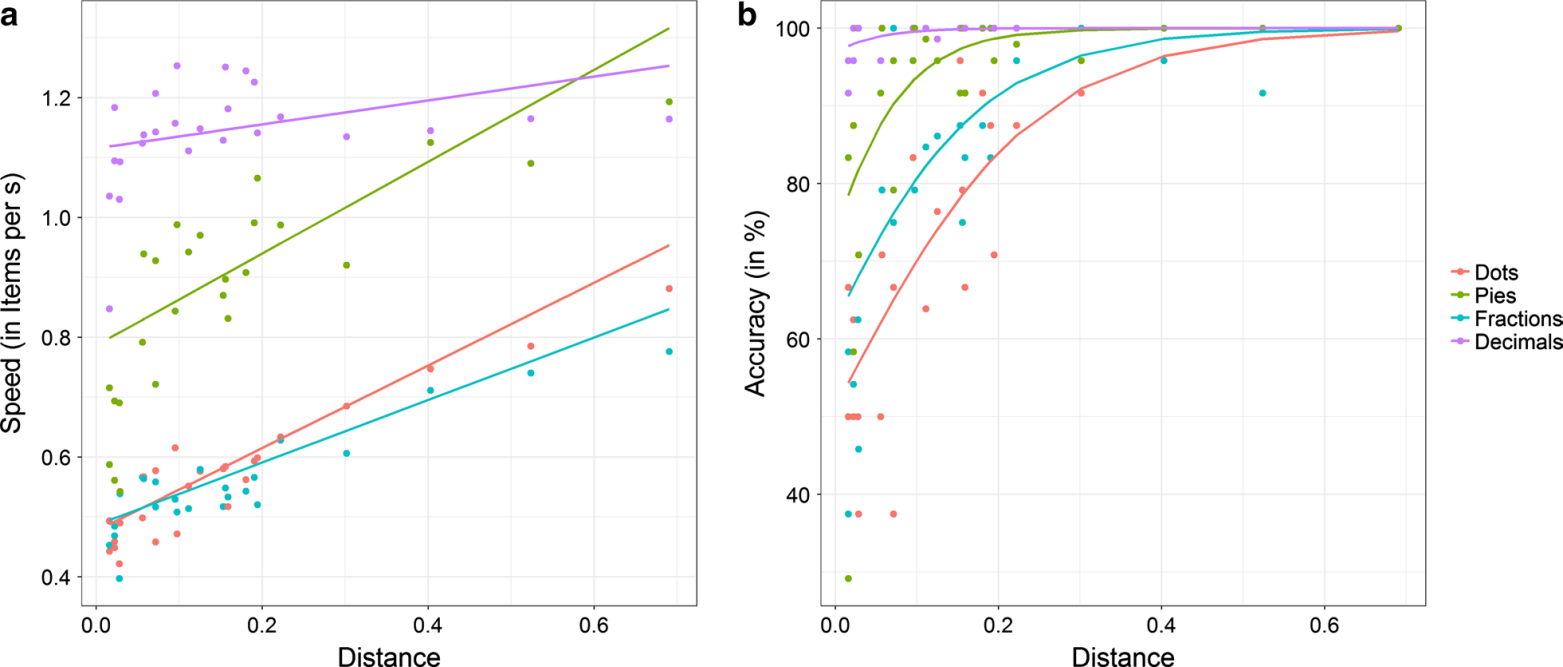
Distance effects in the four conditions (dot patterns, pie charts, fractions, and decimals) for (**a**) speed and (**b**) accuracy. Accuracy was calculated by transforming log odds into percentages

In the next step, we ran a LME with condition, distance between proportions as well as their interaction as fixed effects and speed as dependent variable testing for statistical significance of these differences. All three *F* tests were highly significant [condition: *F*(3, 27.97) = 112.77, *p* < .001, distance: *F*(1, 49.81) = 133.76, *p* < .001, and condition × distance: *F*(3, 30.48) = 20.06, *p* < .001]. This indicated that participants’ speed differed between conditions. Pairwise post hoc comparisons revealed that except for the difference between fractions and dot patterns (*p* = .567) speed in all conditions differed significantly from each other (all *p* < .001). Additionally, the significant distance indicated that across all conditions speed increased with the overall numerical distance, slope = .55 items/sec (*SE* = .05). However, the significant interaction indicated that distance effects varied between conditions. Mean distance effects (*SE* in parenthesis) in the separate conditions were for fractions: .52 (.07) items/sec, *z* = 7.42, *p* < .001, for decimals: .20 (.05) items/sec, *z* = 3.85, *p* < .001, for pies: .77 (.07) items/sec, *z* = 10.39, *p* < .001, and for dots: .69 (.09) items/sec, *z* = 7.40, *p* < .001, respectively. This indicated that we observed significant distance effects in all four presentation formats. Post-hoc analyses indicated that the distance effect for decimals differed significantly from distance effects of all other presentation formats (*p* < .001). Moreover, distance effects of fractions and pie charts differed significantly from each other (*p* = .013). Other pairwise comparisons were not significant (*p* > .139). Figure 3a gives an overview of the distance effects for speed data.

We also evaluated performance differences in accuracy between conditions by running a GLME with the same factors. Again, all three LRT for fixed effects were significant [condition: *χ*^2^(3) = 210.64, *p* < .001, distance: *χ*^2^(1) = 100.75, *p* < .001, and condition × distance: *χ*^2^(3) = 10.28, *p* = .016]. Estimated log odds (SE in parenthesis) of the four conditions were for fractions: 1.96 (.19), in  % = 87.6%, for decimals: 6.63 (1.26), in  % = 99.9%, for pie charts: 3.641 (.34), in  % = 97.4%, and for dots: 1.30 (.17), in  % = 78.6%, respectively. Pairwise post hoc comparisons revealed that log odds of all conditions differed from each other significantly (all *p* < .021). Moreover, the significant distance effect indicated that participants’ accuracy increased with the overall numerical distance between two proportions, slope in log odds = 13.69, *SE* = 2.87. However, again the significant interaction between condition and distance indicated that distance effects differed between conditions. Mean distance effects in log odds (*SE* in parenthesis) in the separate conditions were for fractions: 9.37 (1.60), *z* = 5.86, *p* < .001, for decimals: 20.57 (10.52), *z* = 1.96, *p* = .051, for pie charts: 16.75 (3.02), *z* = 5.55, *p* < .001, and for dots: 8.05 (1.25), *z* = 6.46, *p* < .001, respectively. Pairwise post hoc comparisons revealed that only distance effects for dot patterns and pie charts differed significantly (*p* = .033), whereas all other comparisons were not significant (all *p* > .067). Distance effects for different conditions are shown in Fig. 3b.

### Imaging results

#### Evaluating magnitude processing in different presentation formats

##### Fractions

**Fig. 4:**
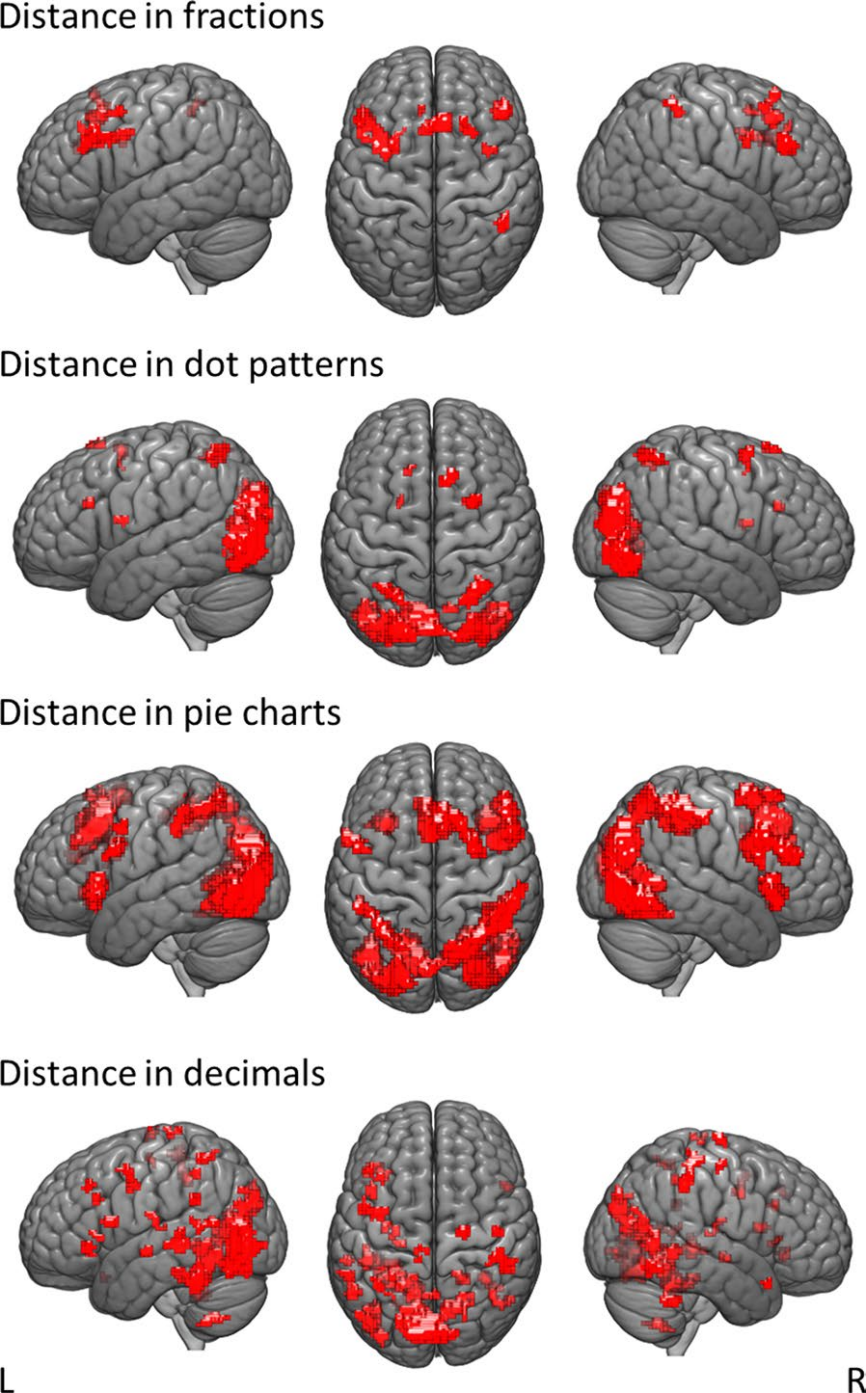
Significant patterns of activation found for distance in the four presentation formats fractions, dot patterns, pie charts, and decimals

Numerical distance in fraction processing was associated with significantly increasing activation in right IPS, bilateral SMA and bilateral frontal gyrus for decreasing distance (see Table 1 and Fig. 4).

**Table 1:** Distance effect in proportion magnitude comparison for different presentation formats

Contrast	Brain region	MNI (x, y, z)			*k*	*t*
Fractions	RH inferior parietal lobule (hIP2)	43	− 42	53	31	5.43
	RH inferior parietal lobule (hIP3)^a^	46	− 45	55		
	RH supplementary motor area	8	23	45	101	7.59
	LH supplementary motor area^a^	− 7	18	48		
	LH middle frontal gyrus	− 47	28	33	176	6.58
	LH inferior frontal gyrus^a^	− 40	21	33		
	LH middle frontal gyrus	− 27	8	50	29	5.92
	RH precentral gyrus	36	3	30	23	5.73
	LH superior medial gyrus	− 7	33	40	11	5.66
	RH inferior frontal gyrus	46	31	25	90	6.98
	RH superior frontal gyrus	21	21	55	34	5.90
Dot patterns	RH superior parietal lobule (hIP3)	33	− 52	60	94	6.05
	LH superior parietal lobule	− 30	− 57	63	73	6.37
	RH superior frontal gyrus	26	3	63	41	6.54
	LH anterior cingulate gyrus	− 15	26	28	13	5.97
	RH calcarine gyrus	12	− 70	18	13	5.27
	LH caudate	− 20	6	18	12	5.69
	LH calcarine gyrus	− 17	− 75	10	1953	7.75
	LH middle occipital gyrus^a^	− 42	− 80	0		
	LH cuneus^a^	− 2	− 75	18		
	RH inferior occipital gyrus^a^	43	− 75	− 8		
Pie charts	RH middle occipital gyrus	28	− 75	33	2094	9.69
	RH superior occipital gyrus^a^	26	− 75	38		
	RH inferior occipital gyrus^a^	43	− 75	− 5		
	RH inferior temporal gyrus^a^	46	− 80	− 3		
	RH inferior parietal lobule (hIP2)^a^	41	− 40	48		
	RH superior parietal lobule (hIP3)^a^	28	− 60	60		
	LH superior parietal lobule	− 22	− 65	63	247	6.62
	LH inferior parietal lobule (hIP3)^a^	− 35	− 50	53		
	RH middle cingulate cortex	8	16	45	836	11.46
	LH middle cingulate cortex^a^	− 5	18	45		
	RH precentral gyrus	46	6	28	532	9.32
	RH inferior frontal gyrus^a^	48	28	25		
	RH insula	36	21	3	397	8.93
	RH inferior frontal gyrus^a^	33	26	− 5		
	LH insula	− 32	18	3	204	9.36
	LH inferior frontal gyrus	− 60	11	25	72	6.41
	LH precentral gyrus	− 45	1	40	37	5.43
	LH inferior occipital gyrus	− 42	− 75	− 10	1086	11.79
	LH middle occipital gyrus^a^	− 42	− 85	8		
	LH superior occipital gyrus^a^	− 25	− 80	25		
	LH calcarine gyrus	− 15	− 72	10	706	8.68
	RH calcarine gyrus^a^	16	− 67	13		
Decimals	RH inferior parietal lobule (hIP2)	48	− 40	45	95	5.96
	RH postcentral gyrus^a^	43	− 32	60		
	LH inferior parietal lobule (hIP3)	− 37	− 52	58	21	5.41
	LH superior parietal lobule^a^	− 30	− 57	63		
	LH inferior parietal lobule	− 27	− 45	48	12	5.45
	LH supramarginal gyrus	− 60	− 45	30	24	5.59
	LH lingual gyrus	− 15	− 55	− 10	468	7.38
	LH fusiform gyurs^a^	− 37	− 37	− 23		
	RH inferior temporal gyrus	51	− 62	− 10	50	5.83
	RH fusiform gyrus^a^	41	− 57	− 13		
	LH inferior occipital gyrus	− 45	− 75	− 13	344	7.60
	LH middle occipital gyrus^a^	− 50	− 75	− 3		
	LH middle temporal gyrus^a^	− 52	− 70	13		
	LH Superior temporal gyrus	− 42	− 35	3	60	7.05
	LH middle temporal gyrus	− 55	− 55	15	45	5.72
	LH superior temporal gyrus^a^	− 57	− 45	15		
	RH temporal pole	51	16	− 23	15	5.89
	LH middle temporal gyrus	− 57	− 37	8	14	5.50
	LH precentral gyrus	− 40	− 2	40	59	5.81
	LH inferior frontal gyrus	− 45	28	− 3	47	5.56
	LH inferior frontal gyrus	− 42	13	15	29	6.66
	RH superior frontal gyrus	21	− 15	75	25	6.21
	RH precentral gyrus	43	− 17	58	16	5.32
	LH Middle frontal gyrus	− 45	26	40	16	5.81
	LH middle frontal gyrus	− 35	21	30	12	5.71
	LH posterior insula	− 30	− 20	13	28	5.72
	LH insula	− 35	21	3	10	5.17
	LH cuneus	− 2	− 77	18	742	7.51
	LH calcarine gyrus^a^	− 15	− 72	13		
	LH superior occipital gyrus^a^	− 12	− 80	23		
	RH lingual gyrus	18	− 47	− 3	43	6.60
	LH putamen	− 30	− 12	− 8	18	5.71
	LH paracentral lobule	− 10	− 32	75	13	5.11

Activations were thresholded at a whole-brain FWE-corrected *p* value of < .05 with a cluster size of *k* = 10 voxels and reported only when they remained significant following FWE-correction for multiple comparisons at the cluster-level at *p* < .05 FWE. Cerebellar activations are not reported due to incomplete coverage of the cerebellum depending on individual head size

*k* cluster size; *LH* left hemisphere; *MNI* Montreal Neurological Institute; *RH* right hemisphere; *t* t value

^a^Minor maximum

##### Dot patterns

Numerical distance in processing dot patterns was associated with activation in bilateral IPS, left ACC, right SFG as well as visual cortex such as left middle and right inferior occipital gyrus with decreasing distance (see Table 1 and Fig. 4).

##### Pie charts

Numerical distance in the processing of pie charts revealed activation in bilateral IPS, large bilateral occipital regions extending to parietal and temporal areas and bilateral IFG with decreasing distance. Further activation was observed in bilateral insula, bilateral precentral gyrus and bilateral MCC (Table 1 and Fig. 4).

##### Decimals

Numerical distance in decimal processing was associated with activation in bilateral IPS, left occipito-temporal regions, left fusiform gyrus and frontal areas with a left-lateralized dominance with decreasing distance. Further clusters of activated voxels were observed in left insula and bilateral precentral gyrus (see Table 1 and Fig. 4).

#### Specific correlates of symbolic and non-symbolic proportional magnitudes

Additionally, an exploratory analysis of specific activations associated with processing symbolic and non-symbolic proportional magnitudes was conducted. Because the activation for theses contrasts did not survive FWE-correction on a whole-brain level, activations were thresholded at a whole-brain p value of < .001 uncorrected and only reported when they remained significant for multiple comparisons at the cluster-level at p < .05 FWE-corrected. This analysis revealed the following results.

##### Symbolic vs. non-symbolic magnitudes

**Fig. 5:**
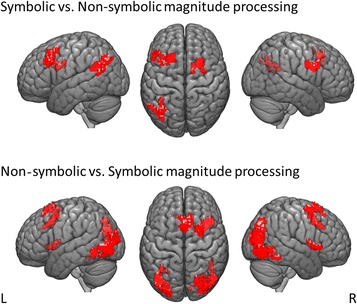
Activations found for the contrast of distance in symbolic vs. non-symbolic and non-symbolic vs. symbolic presentation formats

Distance in processing of symbolic (i.e., fractions and decimals) versus non-symbolic magnitudes (i.e., pie charts and dot patterns) indicated higher activation in bilateral middle frontal gyrus, left SFG, right SMA and left AG (see Table 2 and Fig. 5) with decreasing numerical distance.

**Table 2:** Activations for distance in symbolic vs. non-symbolic as well as non-symbolic vs. symbolic presentation formats

Contrast	Brain region	MNI (x, y, z)			*k*	*t*
Symbolic vs. non-symb.	LH superior frontal gyrus	− 20	21	43	286	6.45
	LH middle frontal gyrus^a^	− 50	23	33		
	LH angular gyrus	− 37	− 60	28	273	4.59
	LH middle occipital gyrus^a^	− 42	− 72	33		
	RH supplementary motor area	21	13	33	229	6.77
	RH middle frontal gyrus^a^	26	8	28		
Non-symb. vs. symbolic	RH inferior temporal gyrus	48	− 72	− 5	1046	6.05
	RH middle occipital gyrus^a^	33	− 75	15		
	RH superior parietal lobule^a^	21	− 72	43		
	LH middle occipital gyrus	− 40	− 75	3	587	5.47
	RH middle cingulate cortex	8	13	43	390	5.76
	RH insula	43	23	0	221	4.54
	RH putamen^a^	28	3	10		
	RH caudate nucleus^a^	16	13	8		
	RH superior frontal gyrus	23	6	63	132	5.31

Activations were thresholded at a whole-brain *p* value of < .001 uncorrected with a cluster size of *k* = 10 voxels and reported only when they remained significant following FWE-correction for multiple comparisons at the cluster-level at *p* < .05 FWE-corrected. Cerebellar activations are not reported due to incomplete coverage of the cerebellum depending on individual head size

*k* cluster size; LH left hemisphere; *MNI* Montreal Neurological Institute; RH right hemisphere; *t t* value

^a^Minor maximum

##### Non-symbolic vs. symbolic magnitudes

Numerical distance in non-symbolic versus symbolic magnitudes was associated with higher activations in a widespread temporal network extending to parietal and occipital cortex, left middle occipital gyrus, right MCC, insula and SFG (see Table 2 and Fig. 5).

#### Conjunction analysis of the distance effect

**Fig. 6:**
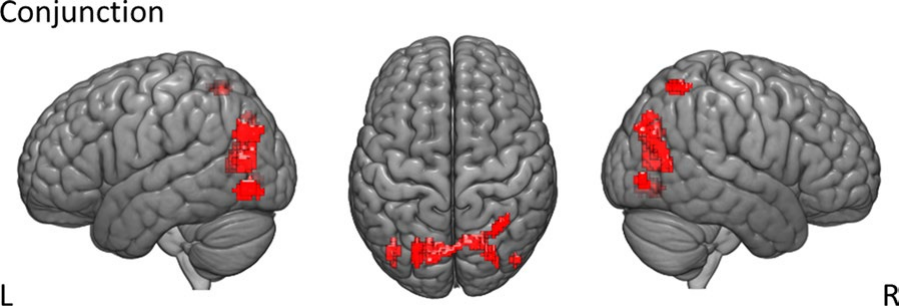
Significant joint activation across the four conditions for distance (e.g., fractions, decimals, dot patterns, pie charts)

As previous studies evaluated shared neural correlates of magnitude processing for fractions and fraction words, we conducted a conjunction analysis ([41, 42]; conjunction null, see [59]) to evaluate the hypothesis of a common neural correlate of magnitude processing for symbolic and non-symbolic proportions. The conjunction analysis revealed significant joint activation in right SPL (hIP3) as well as bilateral occipital regions (see Table 3 and Fig. 6).

**Table 3:** Joint activations across the four conditions (i.e., fractions, decimals, dot patterns, pie charts) for distance as revealed by the conjunction analysis

Contrast	Brain region	MNI (x, y, z)			*k*	*t*
Conjunction	RH superior parietal lobule (hIP3)	31	− 60	60	46	4.50
	LH calcarine gyrus	− 17	− 75	13	276	5.55
	RH calcarine gyrus^a^	16	− 67	18		
	LH cuneus^a^	− 2	− 75	20		
	RH superior occipital gyrus^a^	23	− 75	28		
	LH inferior occipital gyrus	− 40	− 72	− 8	76	4.76
	LH superior occipital gyrus	− 25	− 70	30	73	4.60
	RH middle occipital gyrus	46	− 82	0	22	3.88
	RH inferior occipital gyrus^a^	43	− 80	− 3		

Activations were thresholded at a whole-brain *p* value of < .001 uncorrected with a cluster size of *k* = 10 voxels. Cerebellar activations are not reported due to incomplete coverage of the cerebellum depending on individual head size

*k* cluster size; *LH* left hemisphere; *MNI* Montreal Neurological Institute; RH right hemisphere; *t t* value

^a^Minor maximum

## Discussion

The present study aimed at investigating whether the processing of symbolic and non-symbolic proportions draws on a common underlying neural substrate. Recent neuroimaging evidence indicated that symbolic fractions [41, 42] and non-symbolic proportions [43] are processed within a fronto-parietal network including the IPS. Synced with evidence on whole number processing (e.g., [10, 13] for overviews) this suggests that both absolute and relative magnitude information seem to be processed within this brain area. Nevertheless, a systematic evaluation of brain areas jointly activated when processing symbolic *and* non-symbolic proportion magnitude was missing so far. Therefore, we systematically evaluated the neural correlates of processing symbolic fractions and decimals as well as non-symbolic dot patterns and pie charts in the same experiment. Most importantly, we observed evidence for a common neural substrate in right IPS as well as bilateral visual cortex for processing relative magnitude irrespective of presentation format. In the following, we will first discuss this joint activation found for symbolic and non-symbolic proportions before addressing distance-related activation observed for symbolic and non-symbolic formats and in each presentation format separately.

### A common neural substrate for processing relative magnitude

We observed a common neural substrate for processing symbolic and non-symbolic proportions in an occipito-parietal network comprising the right IPS. Within this network, IPS activation seems to reflect processing of abstract relative magnitude (e.g. [9, 16, 34, 37]), whereas activation in occipital areas might rather reflect higher order visual processing as well as decoding of the visual form [15, 18, 21], which helps to process semantic features of quantity.

Recent research revealed a right-hemispheric preference for the processing of absolute number magnitude [9, 31, 32]. As such, the right IPS seems to specifically underlie the semantic representation of numerical distances [61]. This right-hemispheric preference for the processing of magnitude was reported for both symbolic and non-symbolic quantities (e.g., [8]). Importantly, our data showed joint activation for magnitude processing of symbolic and non-symbolic proportions in an occipito-parietal network including the right IPS. Thus, besides absolute magnitude also relative magnitude information seems to be processed in right IPS, irrespective of presentation format. Importantly, this seems to reflect a neural correlate of an abstract concept for relative magnitude. This is in line with propositions of the triple code model of numerical cognition that numerical magnitude and mental arithmetic are represented and processed within the IPS [10, 13, 62, 63]. Importantly, recent evidence suggested that the respective parietal cortex areas might subserve an abstract, notation-independent representation for both absolute and relative magnitude ([9, 41–43, 64], but see [65] for a more detailed discussion of this point). The results of our conjunction analysis support this assumption. Moreover, our data also extended previous research on proportion processing because so far only the processing of either symbolic fractions [41], fractions and fraction words [42] or non-symbolic proportions [43] was investigated on the neural level. In the present study, we systematically investigated common neural activation for the processing of both symbolic and non-symbolic formats. Our results are also in line with recent research suggesting that humans (and animals) are not necessarily born with a “sense of number”—the ability to perceive, manipulate and understand discrete numerosities [66–68]—but rather a generalized and abstract “sense of magnitude” for the processing of both, numerosities and continuous magnitudes (e.g., size, area, and density; for a review, see [69]). As the present study found a shared neural correlate for both discrete (e.g., fractions and dot patterns) as well as continuous relative magnitudes (e.g., decimals and pie charts) in symbolic and non-symbolic presentation formats, the results further support the idea of such a generalized magnitude system.

Additionally, we found activation in bilateral visual cortex (bilateral superior, bilateral inferior, right middle occipital gyrus). These brain regions are involved in higher order visual processing and decoding of the visual form [15, 18, 21]. Furthermore, the ventral visual stream is anchored in the lateral occipital cortex (LOC; [19]). This stream plays an important role in number representation and magnitude manipulation as it interacts with the IPS for the semantic representation and procedural manipulation of quantity [19]. Importantly, the ventral visual stream areas in the occipital gyrus are not only co-activated with the IPS during numerical and arithmetic processing, but their activation also increases with task complexity [19, 70, 71]. Hence, our data point to higher order visual processing during relative magnitude processing in the ventral visual stream which may reflect the complexity of accessing relative magnitude information.

This task complexity might also be reflected by the distance effect, an effect that is often associated with task difficulty as difficulty increases when the distance between two to-be-compared numbers decreases [11]. The distance effect for symbolic and non-symbolic proportions was observed in the behavioral data. We found differences in distance effects for error rates and reaction times between the different presentation formats. While error rates and reaction times were highest for fractions and dot patterns, comparing pie charts—and even more so decimals—led to faster and more accurate responses. Increasing response times and error rates might reflect influences of task difficulty. Therefore, behavioral data seemed to indicate that accessing magnitude information of proportional relations might not be the only mechanisms involved. This is also reflected in the neural data.

The IPS was previously associated with tasks requiring specific attention due to higher levels of difficulty [72–74]. In studies on number processing the distance effect is a prominent paradigm (e.g., [9, 41]). However, this effect is strongly modulated by difficulty: as the distance between two numerals decreases, error rates as well as response times, and hence, difficulty increases. Thus, the observed activation in IPS during numerical tasks might also be driven by task difficulty. Yet, previous studies found activations in the IPS for either passive listening to number words [75] or passive viewing of symbolic numbers versus letters and colors [37]. Therefore, we are confident that particularly activation in right intraparietal regions, as observed in the present conjunction analysis, reflects processing of (relative) magnitude information over and beyond influences of task difficulty.

### Specific activations for symbolic and non-symbolic presentation formats

The contrast between symbolic (i.e., fractions and decimals) and non-symbolic presentation formats (i.e., dot patterns and pie charts) indicated activation in a fronto-parietal network comprising left AG, left superior and middle frontal gyrus, and right SMA. In line with previous research, activation found for symbolic vs. non-symbolic proportions showed a left-lateralized preference [34].

The left AG has been previously associated with verbally-mediated processes such as the retrieval of arithmetic facts and symbolic numerical processing [10, 28, 29, 36]. Holloway and colleagues [36] argued that the left AG mediates the mapping between a visual form and its semantic referent, that is, between numerical symbols and their magnitudes. However, recent research indicated that the AG plays a more domain-general attentional role that may not be specific to math fact retrieval [30, 76]. In particular, Bloechle and colleagues [30] proposed that the left AG adjusts and adapts relative attentional demands in the neural networks associated with fact retrieval and magnitude manipulation. For accessing magnitude information of decimals a symbol-to-referent mapping in the left AG seems plausible. However, accessing the magnitude of a fraction might require additional computational steps. This might involve increased attentional effort or more demanding symbol-to-referent mapping during the decoding of several numerals of the fraction itself. Thus, the role of the left AG in our data might reflect both scenarios—higher attentional demands or symbol-to-referent mapping.

Furthermore, activation of the left SFG and right SMA may be assumed to reflect goal creation, procedural steps as well as the generation of strategies for solving multi-step problems during the processing of symbolic proportions [13].

In contrast, processing non-symbolic proportions indicated specific activation within the ventral visual stream (bilateral middle occipital gyrus, right inferior temporal gyrus, and right superior parietal lobule), right insula, left MCC, and right SFG. The large cluster of bilateral occipital activation might reflect higher visual demands of the non-symbolic presentation formats. Furthermore, activation of the ventral visual stream might point to the involvement of visuo-spatial functions and covert shifts of attention during processing non-symbolic proportions [19]. In particular, the superior parietal lobe was repeatedly reported for non-symbolic number processing [8, 33, 36, 38] and suggested to host a visual-spatial representation of quantity [21].

Moreover, higher activation of areas associated with cognitive control comprising, amongst others, MCC and SFG might indicate that accessing magnitude information of non-symbolic proportions required stronger involvement of cognitive control processes and performance monitoring than of symbolic proportions [77]. Furthermore, we suggest that the involvement of the SFG might reflect the application of strategies for solving multi-step problems [13]. Together with higher activation of areas subserving cognitive control, the right insula was suggested to be involved in initiating motivated behavior [78], execution of responses [79], and error processing ([80]; see also [13]).

### Distance-related activation = magnitude-related representation?

We also evaluated magnitude-related activation in proportion processing by specifically focusing on the neural correlates of distance in the respective presentation formats. Our findings indicated that processing relative magnitude of symbolic and non-symbolic proportions might not exclusively reflect domain-specific magnitude-related processing. Rather, our results suggest that the idea of a unique reflection of (relative) magnitude processing by distance may be too simplistic. In fact, magnitude processing of proportions might not only reflect specific processing of magnitude information, but may also reflect influences of other less domain-specific cognitive processes involved in distance-related processing. In particular, it seems that different presentation formats contain different cognitive components to different degrees. In the following, we will discuss these differing components as indicated by observed activation of associated brain areas in the current study.

#### Activation in (intra)parietal cortex

Bilateral IPS was repeatedly reported active for processing absolute magnitude [9, 10, 16, 37]. In line with this idea, we found that magnitude processing of *decimals* was associated with activation in the bilateral IPS, most probably reflecting the processing of number magnitude information [8, 61, 64]. In fact, magnitude processing of decimals is very similar to processing absolute magnitude because skipping the leading 0 and just comparing the digits following the decimal point leads to a correct result [64]. Thus, no computation of part-whole relations, and thus, relative magnitude is necessary to access magnitude information of decimals compared to the other presentation formats used in this study. Consequently, the involvement of intraparietal regions typically involved in processing absolute magnitude comes as no surprise.

In line with previous research, our data also revealed activation in right IPS for the magnitude processing of *fractions* [41]. Because the right IPS was repeatedly reported to be activated during absolute number magnitude processing in number comparison tasks [8, 31, 32, 61], our data on the processing of relative magnitude extend these previous findings. In particular, our results indicate that in addition to absolute numerical magnitude, relative magnitude of symbolic proportions is also processed in the IPS. This is significant because additional computational steps may be necessary to access magnitude information of proportions. These findings, thus, further support previous results suggesting that the right IPS is systematically involved in the processing of number magnitude, regardless of number format [8] or notation [9].

For the magnitude processing of *dot patterns* we found activation in the bilateral SPL extending to the IPS in the right hemisphere, which has been repeatedly reported for non-symbolic number processing [8, 33, 36, 38]. While activation of the right IPS might indicate additional computations of part-whole relations necessary for accessing relative magnitude information, activation of bilateral SPL rather reflects the involvement of visuospatial functions such as saccades and covert shifts of attention [19]. This finding seems plausible for this presentation format because eye-movements and attention shifts are particularly necessary to capture discrete quantities and the part-whole relation reflected by proportional dot patterns.

Moreover, we found activation in bilateral IPS and SPL for the magnitude processing in *pie charts*. It has been shown that bilateral IPS is activated during estimation strategies and approximation processes for symbolic and non-symbolic presentation formats [34, 81, 82]. To a certain degree, activation in bilateral superior and inferior parietal lobes might also indicate the involvement of mental rotation strategies [83, 84]. Thus, parietal activation might reflect an additional distance effect caused by the angular degrees between the to-be-compared blue parts of the pie charts. However, as we found joint activation for all presentation formats in right IPS (with the other three formats not requiring mental rotation), parietal activation in magnitude processing of pie charts should reflect not only mental rotation strategies, but at least partially the processing of relative magnitude information as well. This might indicate the involvement of estimation, approximation and mental rotation strategies during accessing relative magnitude information for this specific presentation format.

#### Activation in frontal cortex areas

Furthermore, we observed activation in bilateral inferior and middle frontal gyrus as well as SMA for the magnitude processing of *fractions*. Activation in frontal areas is commonly associated with rather domain-general supplementary executive processes such as strategy choice and procedural planning in numerical cognition [29, 62, 85]. Furthermore, increasing demands on working memory, performance monitoring, goal-directed problem solving, and interference control loads were associated with neural activation in a network comprising these frontal brain regions including IFG, ACC, MCC, and insula [18, 86, 87]. Importantly, the insular-cingulate salience network which initiates control signals during arithmetic problem solving is anchored in the anterior insula and ACC [19, 88]. In line with this rationale, participants may have applied different strategies which involve executive processes for accessing relative magnitude information of fractions. Observed activation in frontal areas might reflect increasing demands on such executive processes during magnitude computation of fractions, and thus, might reflect the active magnitude computation of the given part-whole relation. These computations can be very demanding, and thus, lead to high loads on executive functions. Hence, activation of frontal areas might reflect aspects of difficulty in actually computing relative fraction magnitude and, consequently, additional computational strategies necessary for doing so.

Importantly, it was shown that context-dependent shifts in strategy might also cause differences in activation of frontal brain regions [89]. Thus, frontal areas might be activated differently according to which strategy is applied for accessing relative magnitude information for the respective proportion and how high the demand on executive functions actually is. This is also reflected by the results of our conjunction analysis as we did not find joint frontal activation for all presentation formats. However, each presentation format elicited separate activation in specific frontal brain regions. Yet, these brain regions apparently did not overlap. Hence, different strategies seemed to be applied for accessing magnitude information of the respective presentation formats which, in turn, may have led to distinct activations in frontal areas.

Magnitude processing of *decimals* elicited activation in IFG, MFG and insula exclusively in the left hemisphere. IFG is typically involved in processing simple numerical tasks with low working memory or procedural requirements, while MFG and insula rather tend to support working memory systems and goal-directed attention maintenance [13, 18, 23].

For *pie charts*, computations of part-whole relations and visual strategies might play a crucial role for accessing relative magnitude information. Again, applying these visual estimation strategies and computations might have led to increased working memory demands as reflected by activation of bilateral IFG [90, 91]. Thus, activation in bilateral IFG, bilateral MCC as well as bilateral insula observed for magnitude processing of *pie charts* may indicate the involvement of the salience network as well as working memory and goal-directed attention processes also in this presentation format [19, 88].

We observed activation in left ACC and the right SFG for the magnitude processing of *dot patterns*. These activations indicated specific demands on working memory and cognitive control when accessing magnitude information of proportional dot patterns [13, 41]. In particular, the involvement of the SFG may further reflect the generation of strategies for solving multi-step problems [13]. Hence, to access magnitude information of proportional dot patterns, participants seem to apply multi-step strategies for summation and quantification of non-symbolic part-whole relations, which in turn lead to increased working memory and cognitive control demands.

#### Activation of occipital brain areas

Previous studies showed that high attentional loads in visual processing, encoding, and reanalysis, as well as visual manipulations evoke activations in occipital areas [16, 92, 93]. We found activation in these brain regions for magnitude processing of both *pie charts* as well as *dot patterns*. Thus, accessing magnitude information of pie charts seems to recruit a wide range of executive processes and visual strategies, which are associated with a wide range of brain activations in fronto-parietal and occipital regions.

Magnitude processing of *dot patterns* was associated with activation of occipital brain regions involved in processing visual information. This activation in bilateral occipital gyri, thus, might reflect the specific processing demands on visual information to access magnitude information in this presentation format. Activation in visual cortex might be even stronger when participants drive their attention to a specific object, i.e. proportional dot patterns or pie charts [94]. Thus, to derive relative magnitude information of dot patterns and pie charts participants seemed to strongly rely on visual strategies. In particular, accessing magnitude information for non-symbolic proportions might be associated with increasing visual processing demands, and thus, with increasing activation in visual areas.

Furthermore, we also found activation in the left occipital gyrus extending to fusiform gyrus, occipitotemporal areas as well as middle and superior temporal gyrus associated with magnitude processing of *decimals*. Interestingly, the ventral visual stream areas consisting, amongst others, of lateral occipital cortex, fusiform gyrus and inferior temporal cortex were co-activated with the IPS during arithmetic processing [19]. In this context, activation in occipitotemporal regions including the fusiform gyrus might indicate the involvement of the visual number form area during magnitude processing [62]. Although speculative, an explanation for this finding might be that participants had to visually encode more digits in trials with smaller distance. The smaller the distance, the further to the right in the digit string the decisive digit is to be found (e.g., .24_.75 vs. .53_.56). Thus, more digits had to be encoded visually to access the respective magnitude information. Visually encoding digits might in turn have led to increased activation in the visual number form area and the occipital gyrus. Additionally, activation in left superior temporal regions, which are typically involved in reading processes [95, 96], may reflect the connection between numerical symbols and their quantitative referents [36]. Interestingly, however, we failed to find activation in the visual number form area associated with the magnitude processing of fractions. This might indicate that the difficulty of this specific presentation format and cognitive demands during the additional computational steps for accessing magnitude information of fractions might be predominant over visual encoding processes. Furthermore, although previous studies suggested that fractions are represented holistically in the human brain [41], our results suggest that access to the magnitude information of a fraction seems to involve additional computational steps as reflected by activation of frontal working memory and cognitive control areas rather than a simple symbol-to-referent mapping. This might also explain why we did not observe any activation in the visual number form area for magnitude processing of fractions.

### Practical implications of our study

From a more practical perspective, the neurocognitive results presented here might indicate that a shared use of symbolic and non-symbolic presentation formats could be supportive for teaching and learning fractions because they activate a joint neural correlate reflecting abstract relative magnitude processing. Moreover, it is known that learning with multiple representations can enhance students’ understanding of new concepts [97]. However, teaching fractions currently focuses strongly on memorization of procedures and not on conceptual understanding [98, 99]. Yet, in order to choose the appropriate procedure to solve fraction problems, these procedures should be underpinned by good conceptual knowledge about fractions [100]. An intervention study of Gabriel and colleagues [6] showed that the use of non-symbolic presentation formats to represent and manipulate fractions improved students’ conceptual understanding of fractions and their magnitudes. Additionally, an intelligent tutoring system as described by Rau and colleagues [4] enhanced the conceptual understanding of fraction magnitude by specifically associating symbolic fractions with non-symbolic presentation formats. After working with this tutoring system as part of their regular mathematics instructions, 4th- and 5th-grade students improved significantly in their conceptual understanding of fractions (for an overview, see [99]).

Although speculative, these positive effects when jointly using symbolic and non-symbolic presentation formats for teaching conceptual understanding of fractions, might be partly based on a shared neural correlate for relative magnitude processing. However, the benefit of jointly using symbolic and non-symbolic formats might be additionally driven by complementary mechanisms in relative magnitude processing of different presentation formats: in addition to the shared neural correlate, all presentation formats showed distinct and specific activation patterns in the current study, which points to different additional (sub)processes that are linked to each presentation format. Because these (sub)processes differ for all presentation formats, non-symbolic presentation formats might complement symbolic formats and vice versa for conceptual understanding. Thereby, children who do not excel at understanding a particular presentation format might be able to compensate for these difficulties by means of other formats. The processing pathways for the presentation formats seem to differ partially depending on the format but to finally converge to abstract magnitude processing in the right IPS.

Thus, the present findings seem to support previous findings of intervention studies on the conceptual understanding of fractions and proportions from a neurocognitive perspective and vice versa.

## Conclusion

Regions around the IPS are commonly associated with the processing of absolute magnitude (e.g., [8, 9]). However, recent research indicated that also relative magnitude information is associated with activation in parietal brain regions [41–43, 64]. Thus, brain areas involved in processing absolute magnitude of numbers were also activated during processing relative magnitude of symbolic fractions as well as non-symbolic proportions. Here, we investigated systematically whether the processing of symbolic and non-symbolic proportions draws on shared underlying neural correlates. Results of the present study indicated joint activation of specific occipito-parietal areas, including right IPS for both symbolic and non-symbolic proportions. In particular, the right IPS is associated with number magnitude processing [8, 9, 32, 61], while the occipital activation during magnitude processing rather reflects the higher order visual processing, which contributes to building semantic representations of quantity [15, 18, 21]. Thus, our findings indicate a shared neural substrate for a format-independent, abstract concept of relative magnitude.

Yet, our results may also be influenced by task difficulty. Nevertheless, activations in the IPS cannot be attributed to task difficulty exclusively, but also reflected specific processing of relative magnitude information. Furthermore, influences of task difficulty might rather be reflected by observed activation in frontal areas due to increasing demands on executive functions. Interestingly, we did not observe joint frontal activation for all presentation formats although all presentation formats elicited significant activation in frontal brain regions. This might indicate that participants applied different strategies depending on the respective presentation format. For instance, while demands on cognitive control and working memory may be lower for magnitude processing of decimals, magnitude processing of fractions might rather be associated with additional computational steps, and thus, with higher demands on working memory and cognitive control. Furthermore, participants might use estimation strategies for magnitude processing of pie charts whereas summation and quantification strategies might support magnitude processing of dot patterns.

Nevertheless, the present data provide evidence for a shared neural correlate for processing relative magnitude, irrespective of symbolic or non-symbolic presentation format.

## References

[1] Gigerenzer G (2002). Calculated risk: how to know when numbers deceive you.

[2] Siegler RS, Fazio LK, Bailey DH, Zhou X (2013). Fractions: the new frontier for theories of numerical development. Trends Cogn Sci..

[3] NMAP. Foundations for success: the final report of the National Mathematics Advisory Panel. Washington, DC: US Department of Education; 2008.

[4] Rau MA, Aleven V, Rummel N, Rohrbach S. Sense making alone doesn’t do it: fluency matters too! ITS support for robust learning with multiple representations. In: Cerri S, Clancey W, Papadourakis G, Panourgia K, editors. Intell. Tutoring Syst. 7315th ed. Berlin/Heidelberg: Springer; 2012. p. 174–84.

[5] Rau MA, Aleven V, Rummel N (2015). Successful learning with multiple graphical representations and self-explanation prompts. J Educ Psychol..

[6] Gabriel F, Coche F, Szucs D, Carette V, Rey B, Content A (2012). Developing children’s understanding of fractions: an intervention study. Mind Brain Educ..

[7] Nieder A (2005). Counting on neurons: the neurobiology of numerical competence. Nat Rev Neurosci..

[8] Piazza M, Pinel P, Le Bihan D, Dehaene S (2007). A magnitude code common to numerosities and number symbols in human intraparietal cortex. Neuron..

[9] Pinel P, Dehaene S, Rivière D, Le Bihan D (2001). Modulation of parietal activation by semantic distance in a number comparison task. Neuroimage..

[10] Dehaene S, Piazza M, Pinel P, Cohen L (2003). Three parietal circuits for number processing. Cogn Neuropsychol..

[11] Moyer RS, Landauer TK (1967). Time required for judgements of numerical inequality. Nature..

[12] Meert G, Grégoire J, Noël M-P (2009). Rational numbers: componential versus holistic representation of fractions in a magnitude comparison task. Q J Exp Psychol..

[13] Arsalidou M, Taylor MJ (2011). Is 2+2=4? Meta-analyses of brain areas needed for numbers and calculations. Neuroimage..

[14] Bugden S, Price GR, McLean DA, Ansari D (2012). The role of the left intraparietal sulcus in the relationship between symbolic number processing and children’s arithmetic competence. Dev. Cogn. Neurosci..

[15] Jolles D, Supekar K, Richardson J, Tenison C, Ashkenazi S, Rosenberg-Lee M (2016). Reconfiguration of parietal circuits with cognitive tutoring in elementary school children. Cortex..

[16] Pinel P, Piazza M, Le Bihan D, Dehaene S (2004). Distributed and overlapping cerebral representations of number, size, and luminance during comparative judgments. Neuron..

[17] Menon V, Rivera SM, White CD, Glover GH, Reiss AL (2000). Dissociating prefrontal and parietal cortex activation during arithmetic processing. Neuroimage..

[18] Fias W, Menon V, Szucs D (2013). Multiple components of developmental dyscalculia. Trends Neurosci. Educ..

[19] Menon V, Dowker A (2015). Arithmetic in the child and adult brain. Cohen Kadosh R.

[20] Ansari D, Garcia N, Lucas E, Hamon K, Dhital B (2005). Neural correlates of symbolic number processing in children and adults. Neuroreport..

[21] Ansari D (2008). Effects of development and enculturation on number representation in the brain. Nat. Rev. Neurosci..

[22] van Dijck J-P, Gevers W, Fias W (2009). Numvbers are associated with different types of spatial information depending on the task. Cognition..

[23] Majerus S, D’Argembeau A, Martinez Perez T, Belayachi S, Van der Linden M, Collette F (2010). The commonality of neural networks for verbal and visual short-term memory. J. Cogn. Neurosci..

[24] Hitch GJ (1978). Role of short-term working memory in mental arithmetic. Cogn. Psychol..

[25] Cohen Kadosh R, Henik A, Rubinstein O, Mohr H, Dori H, Van de Ven V (2005). Are numbers special? The comparison systems of the human brain investigated by fMRI. Neuropsychologia..

[26] Kaufmann L, Koppelstaetter F, Delazer M, Siedentopf C, Rhomberg P, Golaszewski S (2005). Neural correlates of distance and congruity effects in a numerical Stroop task, an event-related fMRI study. Neuroimage..

[27] Ansari D, Grabner RH, Koschutnig K, Reishofer G, Ebner F (2011). Individual differences in mathematical competence modulate brain responses to arithmetic errors: an fMRI study. Learn. Individ. Differ..

[28] Dehaene S, Spelke ES, Pinel P, Stanescu R, Tsivkin S (1999). Sources of mathematical thinking: behavioral and brain-imaging evidence. Science..

[29] Grabner RH, Ansari D, Koschutnig K, Reishofer G, Ebner F, Neuper C (2009). To retrieve or to calculate? Left angular gyrus mediates the retrieval of arithmetic facts during problem solving. Neuropsychologia..

[30] Bloechle J, Huber S, Bahnmueller J, Rennig J, Willmes K, Cavdaroglu S (2016). Fact learning in complex arithmetic - the role of the angular gyrus revisited. Hum. Brain Mapp..

[31] Chochon F, Cohen L, van de Moortele PF, Dehaene S (1999). Differential contributions of the left and right inferior parietal lobules to number processing. J. Cogn. Neurosci..

[32] Dehaene S (1996). The organization of brain activations in number comparison: event-related potentials and the additive-factors method. J. Cogn. Neurosci..

[33] Piazza M, Izard V, Pinel P, Le Bihan D, Dehaene S (2004). Tuning curves for approximate numerosity in the human intraparietal sulcus. Neuron..

[34] Venkatraman V, Ansari D, Chee MWL (2005). Neural correlates of symbolic and non-symbolic arithmetic. Neuropsychologia..

[35] Sokolowski HM, Fias W, Mousa A, Ansari D (2017). Common and distinct brain regions in both parietal and frontal cortex support symbolic and nonsymbolic number processing in humans: A functional neuroimaging metaanalysis. Neuroimage..

[36] Holloway ID, Price GR, Ansari D (2010). Common and segregated neural pathways for the processing of symbolic and nonsymbolic numerical magnitude: an fMRI study. Neuroimage..

[37] Eger E, Sterzer P, Russ MO, Giraud AL, Kleinschmidt A (2003). A supramodal number representation in human intraparietal cortex. Neuron..

[38] Ansari D, Dhital B, Siong SC (2006). Parametric effects of numerical distance on the intraparietal sulcus during passive viewing of rapid numerosity changes. Brain Res..

[39] Delazer M, Ischebeck A, Domahs F, Zamarian L, Koppelstaetter F, Siedentopf C (2005). Learning by strategies and learning by drill - evidence from an fMRI study. Neuroimage..

[40] Ischebeck A, Koschutnig K, Reishofer G, Butterworth B, Neuper C, Ebner F (2010). Processing fractions and proportions: An fMRI study. Int. J. Psychophysiol..

[41] Ischebeck A, Schocke M, Delazer M (2009). The processing and representation of fractions within the brain. An fMRI investigation. Neuroimage..

[42] Jacob SN, Nieder A (2009). Notation-independent representation of fractions in the human parietal cortex. J. Neurosci..

[43] Jacob SN, Nieder A (2009). Tuning to non-symbolic proportions in the human frontoparietal cortex. Eur. J. Neurosci..

[44] Siegler RS, Fuchs L, Jordan NC, Gersten R, Ochsendorf R. The center for improving learning of fractions: a progress report. In: Chinn S, editor. Routledge Int. Handb. Dyscalculia Math. Learn. Difficulties. New York: Routledge; 2015. p. 292–303.

[45] Rau MA, Aleven V, Rummel N (2013). Interleaved practice in multi-dimensional learning tasks: which dimension should we interleave?. Learn Instr..

[46] Rau MA, Aleven V, Rummel N. Intelligent tutoring systems with multiple representations and self-explanation prompts support learning of fractions. In: Dimitrova V, Mizoguchi R, Du Boulay B, editors. 14th Int. Conf. Artif. Intell. Educ. Amsterdam: IOS Press; 2009. p. 441–8.

[47] Matthews PG, Chesney DL (2015). Fractions as percepts? Exploring cross-format distance effects for fractional magnitudes. Cogn Psychol..

[48] Common Core State Standards Initiative. Common Core State Standards for Mathematics 2010. http://www.corestandards.org/. Accessed cited 6 Mar 2018.

[49] Ratcliff R (1993). Methods for dealing with reaction time outliers. Psychol Bull..

[50] Baayen RH, Davidson DJ, Bates DM (2008). Mixed-effects modeling with crossed random effects for subjects and items. J Mem Lang..

[51] Jaeger TF (2008). Categorical data analysis: away from ANOVAs (transformation or not) and towards logit mixed models. J Mem Lang..

[52] Barr DJ, Levy R, Scheepers C, Tily HJ (2013). Random effects structure for confirmatory hypothesis testing: keep it maximal. J Mem Lang..

[53] R Core Team (2015). R: A language and environment for statistical computing.

[54] Bates D, Maechler M, Bolker B, Walker S. Fitting linear mixed-effects models using lme4. J Stat Softw.67:1–48.

[55] Judd CM, Westfall J, Kenny DA (2012). Treating stimuli as a random factor in social psychology: a new and comprehensive solution to a pervasive but largely ignored problem. J Pers Soc Psychol..

[56] Singmann H, Bolker B, Westfall J. afex: analysis of factorial experiments. R package version 0.2015. p. 13–145.

[57] Hothorn T, Bretz F, Westfall P (2008). Simultaneous inference in general parametric models. Biom J..

[58] Benjamini Y, Hochberg Y (1995). Controlling the false discovery rate: a practical and powerful approach to multiple testing. J R Stat Soc Ser B..

[59] Nichols T, Brett M, Andersson J, Wager T, Poline JB (2005). Valid conjunction inference with the minimum statistic. Neuroimage..

[60] Eickhoff SB, Stephan KE, Mohlberg H, Grefkes C, Fink GR, Amunts K (2005). A new SPM toolbox for combining probabilistic cytoarchitectonic maps and functional imaging data. Neuroimage..

[61] Mussolin C, Noel MP, Pesenti M, Grandin C, De Volder A (2013). Neural correlates of the numerical distance effect in children. Front Psychol..

[62] Dehaene S, Cohen L. Towards an anatomical and functional model of number processing. Math Cogn. 1:83–120.

[63] Dehaene S, Cohen L (1997). Cerebral pathways for calculation: double dissociation between rote verbal and quantitative knowledge of arithmetic. Cortex..

[64] DeWolf M, Chiang JN, Bassok M, Holyoak KJ, Monti MM (2016). Neural representations of magnitude for natural and rational numbers. Neuroimage..

[65] Cohen Kadosh R, Walsh V (2009). Numerical representation in the parietal lobes: Abstract or not abstract?. Behav. Brain Sci..

[66] Dehaene S (1997). The number sense: How the mind creates mathematics.

[67] Cantlon JF, Platt ML, Brannon EM (2009). Beyond the number domain. Trends Cogn. Sci..

[68] Feigenson L, Dehaene S, Spelke E (2004). Core systems of number. Trends Cogn. Sci..

[69] Leibovich T, Katzin N, Harel M, Henik A (2017). From, “sense of number” to “sense of magnitude”: The role of conitnuous magnitudes in numerical cognition. Behav. Brain Sci..

[70] Keller K, Menon V (2009). Gender differences in the functional and structural neuroanatomy of mathematical cognition. Neuroimage..

[71] Rosenberg-Lee M, Tsang JM, Menon V (2009). Smybolic, numeric, and magnitude representations in the parietal cortex. Behav. Brain Sci..

[72] Shuman M, Kanwisher N (2004). Numerical magnitude in the human parietal lobe: tests of representational generality and domain specificity. Neuron..

[73] Culham JC, Brandt SA, Cavanagh P, Kanwisher N, Dale AM, Tootell RB (1998). Cortical fMRI activation produced by attentive tracking of moving targets. J. Neurophysiol..

[74] Culham JC, Kanwisher N (2001). Neuroimaging of cognitive functions in human parietal cortex. Curr. Opin. Neurobiol..

[75] Klein E, Moeller K, Nuerk H-C, Willmes K (2010). On the neuro-cognitive foundations of basic auditory number processing: an fMRI study. Behav. brain Funct..

[76] Rosenberg-Lee M, Chang TT, Young CB, Wu S, Menon V (2011). Functional dissociations between four basic arithmetic operations in the human posterior parietal cortec: A cytoarchitectonic mapping study. Neuropsychologia..

[77] MacDonald AW, Cohen JD, Stenger VA, Carter CS (2000). Dissociating the role of the dorsolateral prefrontal and anterior cingulate cortex in cognitive control. Science..

[78] Uddin LQ, Menon V (2009). The anterior insula in autism: under-connected and under-examined. Neurosci. Biobehav. Rev..

[79] Huettel AS, Guzeldere G, McCarthy G (2001). Dissociating the neural mechanisms of visual attention in charge of detection using functional MRI. J. Cogn. Neurosci..

[80] Hester R, Fassbender C, Garavan H (2004). Individual differences in error processing: A review and reanalysis of three event-related fMRI studies using GO/NOGO task. Cereb. Cortex..

[81] Castelli F, Glaser DE, Butterworth B (2006). Discrete and analogue quantity processing in the parietal lobe: a functional MRI study. Proc. Natl. Acad. Sci. U. S. A..

[82] Piazza M, Mechelli A, Price CJ, Butterworth B (2006). Exact and approximate judgements of visual and auditory numerosity: An fMRI study. Brain Res..

[83] Alivisatos B, Petrides M (1997). Functional activation of the human brain during mental rotation. Neuropsychologia..

[84] Jordan K, Heinze H-J, Lutz K, Kanowski M, Jäncke L (2001). Cortical Activations during the Mental Rotation of Different Visual Objects. Neuroimage..

[85] Klein E, Suchan J, Moeller K, Karnath HO, Knops A, Wood G (2016). Considering structural connectivity in the triple code model of numerical cognition: differential connectivity for magnitude processing and arithmetic facts. Brain Struct. Funct..

[86] Peterson BS, Kane MJ, Alexander GM, Lacadie C, Skudlarski P, Leung H-C, et al. An event-related functional MRI study comparing interference effects in the Simon and Stroop tasks. Cogn. Brain Res. 13:427–40. 10.1016/s0926-6410(02)00054-x 11919006

[87] Lui X, Banich MT, Jacobson BL, Tanabe JL (2004). Common and distinct neural substrates of attentional control in an integrated Simon and spatial Stroop task as assessed by event-related fMRI. Neuroimage..

[88] Supekar K, Menon V (2012). Developmental maturation of dynamic causal control signals in higher-order cognition: a neurocognitive network model. PLoS Comput. Biol..

[89] Wagner AD, Desmond JE, Glover GH, Gabrieli JDE (1998). Prefrontal cortex and recognition memory: functional-MRI evidence for context-dependent retrieval processes. Brain..

[90] Bunge SA, Kahn I, Wallis JD, Miller EK, Wagner AD (2003). Neural circuits subserving the retrieval and maintenance of abstract rules. J. Neurophysiol..

[91] Taillan J, Ardiale E, Anton JL, Nazarian B, Félician O, Lemaire P (2015). Processes in arithmetic strategy selection: a fMRI study. Front. Psychol..

[92] Somers DC, Dale AM, Seiffert AE, Tootell RBH (2006). Functional MRI reveals spatially specific attentional modulation in human primary visual cortex. Proc. Natl. Acad. Sci. U. S. A..

[93] Wood G, Nuerk HC, Willmes K (2006). Neural representations of two-digit numbers: A parametric fMRI study. Neuroimage..

[94] Müller NG, Kleinschmidt A (2003). Dynamic interaction of object-and space-based attention in retinotopic visual areas. J. Neurosci..

[95] Raij T, Uutela K, Hari R (2000). Audiovisual integration of letters in the human brain. Neuron..

[96] Van Atteveldt N, Formisano E, Goebel R, Blomert L (2004). Integration of letters and speech sounds in the human brain. Neuron..

[97] Ainsworth S (2006). DeFT: A conceptual framework for considering learning with multiple representations. Learn. Instr..

[98] Lortie-Forgues H, Tian J, Siegler RS (2015). Why is learning fraction and decimal arithmetic so difficult?. Dev. Rev..

[99] Obersteiner A, Dresler T, Bieck SM, Moeller K. Understanding Fractions: Integrating Results from Mathematics Education, Cognitive Psychology, and Neuroscience. In: Norton A, Alibali MW, editors. Constr. Number - Merging Perspect. from Psychol. Math. Educ. Heidelberg: Springer; 2018.

[100] Swan M. Dealing with misconceptions in mathematics. In: Gates P, editor. Issues Math. Teach. London: Routledge/Falmer; 2001. p. 147–65.

